# Sex-differences in Mediterranean diet: a key piece to explain sex-related cardiovascular risk in obesity? A cross-sectional study

**DOI:** 10.1186/s12967-023-04814-z

**Published:** 2024-01-10

**Authors:** Luigi Barrea, Ludovica Verde, Rosario Suárez, Evelyn Frias-Toral, Celina Andrade Vásquez, Annamaria Colao, Silvia Savastano, Giovanna Muscogiuri

**Affiliations:** 1 Dipartimento di Scienze Umanistiche, Università Telematica Pegaso, Via Porzio, Centro Direzionale, isola F2, 80143 Naples, Italy; 2 Unità di Endocrinologia, Diabetologia e Andrologia, Dipartimento di Medicina Clinica e Chirurgia, Centro Italiano per la cura e il Benessere del Paziente con Obesità (C.I.B.O), Università degli Studi di Napoli Federico II, Via Sergio Pansini 5, 80131 Naples, Italy; 3 Department of Public Health, University of Naples Federico II, Via Sergio Pansini 5, 80131 Naples, Italy; 4 School of Medicine, Universidad Técnica Particular de Loja, Calle París, San Cayetano Alto, Loja, 110107 Ecuador; 5 School of Medicine, Universidad Católica Santiago de Guayaquil, Av. Pdte. Carlos Julio Arosemena Tola, Guayaquil, 090615 Ecuador; 6 Unità di Endocrinologia, Diabetologia e Andrologia, Dipartimento di Medicina Clinica e Chirurgia, Università degli Studi di Napoli Federico II, Via Sergio Pansini 5, 80131 Naples, Italy; 7 Cattedra Unesco “Educazione alla Salute e Allo Sviluppo Sostenibile”, University Federico II, 80131 Naples, Italy

**Keywords:** Obesity, Mediterranean diet, Sex differences, Gender, Cardiovascular risk, High sensitivity C reactive protein, Diet, Nutrition

## Abstract

**Background:**

Mediterranean Diet (MD) has many health benefits, particularly in reducing cardiovascular risk (CVR). However, it is still little known if there are any sex differences in following this nutritional pattern and, thus, the potential sex-related repercussions on CVR in obesity. The study aimed to characterize sex-related adherence to MD and its association with CVR factors in subjects with obesity.

**Methods:**

A total of 968 females (33.81 ± 11.06 years; BMI 34.14 ± 7.43 kg/m^2^) and 680 males (aged 34.77 ± 11.31years; BMI 33.77 ± 8.13 kg/m^2^) were included in a cross-sectional observational study. Lifestyle habits, anthropometric parameters, high sensitivity C-reactive protein (hs-CRP), and adherence to MD were evaluated.

**Results:**

Females had significantly higher adherence to MD and lower hs-CRP levels than males (p < 0.001). Additionally, females consumed significantly more vegetables, fruits, legumes, fish/seafood, nuts, and sofrito sauce and less quantity of olive oil, butter, cream, margarine, red/processed meats, soda drinks (p = 0.001), red wine, and commercial sweets and confectionery than their counterparts. A PREDIMED score of ≤ 6 was associated with a significantly increased CVR in both sexes.

**Conclusions:**

Females had higher adherence to MD, lower CVR, and different food preferences than males. Although the same PREDIMED threshold has been identified as a spy of CVR, the sex-related preference of individual foods included in the MD could explain the different impact of this nutritional pattern on CVR in both sexes.

**Graphical Abstract:**

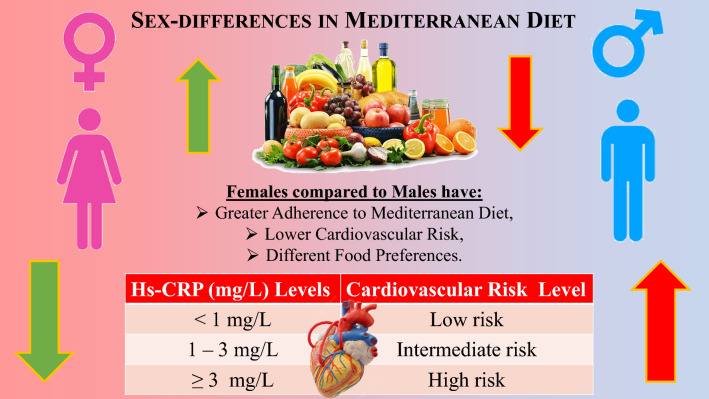

## Introduction

Obesity has become a growing challenge globally, with a significant impact on public health and a wide range of negative consequences for individuals [1]. Worldwide, the incidence of obesity is increasing, with consequences ranging from cardiovascular disease and metabolic disorders to conditions such as certain cancers [2]. Interestingly, in parallel with obesity, a state of inflammation is mediated by the overproduction of reactive oxygen species [3].

One of the critical dimensions of obesity concerns the sex differences that emerge in its prevalence, management, and progression [4, 5]. Indeed, the prevalence of obesity is higher among men than in women, while women have a higher percentage of body fat content compared to men, and sex appears to be an essential factor in the manifestation of central (android) or peripheral (gynoid) obesity. These physiological differences may contribute to the observed sex disparities in the incidence and progression of obesity [4, 5]. In fact, the central (visceral) fat distribution observed in men is associated with negative metabolic consequences, such as insulin resistance, increased free fatty acids, and triglyceride levels [6]. Conversely, the peripheral fat distribution, typically found in women, is generally less associated with such metabolic complications [6].

While there are various modes of intervention to address obesity, lifestyle intervention is the first line of approach to managing this condition [7]. Adopting a balanced and healthy diet, along with increased physical activity, has been shown to be effective in promoting weight loss and improving overall health [7]. However, the lifestyle approach is not fully effective [8], thus suggesting that maybe there could be some issues that are not taken into account.

Indeed, it is well known that sex, along with other non-modifiable and modifiable factors such as age, body weight, or educational status, influence food preferences and consumption amounts [9]. In particular, women’s dietary profiles are characterized by a higher carbohydrate intake, including fruit and vegetables [10]. In contrast, men consume more animal protein-rich diets, including meat, eggs, and dairy products, and less fruit than women, who are more concerned about weight control, food naturalness, and ethical issues [10].

However, little is known regarding sex differences and adherence to specific dietary regimes, such as the Mediterranean diet (MD), widely recognized for its health benefits, including reducing the risk of cardiovascular and metabolic diseases [11]. The food pyramid reflecting the Mediterranean dietary traditions is characterized by a high intake of plant foods (fruits and vegetables), whole grains (including cereals, bread, rice, or pasta, and nuts containing antioxidants; a moderate intake of dairy products (principally cheese and yogurt), fish, particularly fatty fish rich in polyunsaturated fatty acids; a low intake of red meat, processed meats, and sweets; zero to four eggs consumed weekly; extra virgin olive oil (EVOO) as the principal source of fat; and wine consumed in low to moderate amounts, typically with meals [11].

MD has been incorporated into several international and national nutritional guidelines, including those of the World Health Organization (WHO) [12] and the United States Department of Agriculture (USDA) [13]. Nevertheless, it is important to note that adherence to MD can vary significantly between individuals, influenced by several factors, including food preferences, accessibility, and cultural habits [11].

To our knowledge, no study has yet specifically examined sex differences in adherence to MD in subjects with obesity. In a study of 366 adults with ischemic heart disease designed to assess the effect of biological (i.e., sex-related) and psycho-socio-cultural (i.e., gender-related) factors on adherence to MD, male personality traits and perceived stress (i.e. gender identity) were associated with low adherence to MD, regardless of sex, age, and comorbidities, while no statistically significant differences were observed between subjects with medium–high (Body Mass Index (BMI) 27.2 ± 4.5 kg/m^2^) and low adherence (BMI 26.8 ± 4.6 kg/m^2^) to the MD in relation to sex [14].

Physiological and metabolic differences between men and women may result in different nutritional needs, and therefore a one-size-fits-all approach may not be appropriate for both sexes, as it currently occurs.

Therefore, the aim of this study was to characterize in more detail the differences in adherence to MD and its dietary components in female and male subjects with obesity and to investigate their role in sex-related cardiovascular risk (CVR).

## Methods

### Design, setting, and population study

We carried out a cross-sectional observational study at the Endocrinology, Diabetology, and Andrology Unit of the Department of Clinical Medicine and Surgery at Federico II University of Naples. The study took place between January 2015 and January 2023. The data collected from the subjects were entered into an electronic medical record and stored in a large, anonymized database for research purposes after obtaining their signed informed consent.

The inclusion criteria for selecting subjects in the study were as follows:

- BMI ≥ 18.5 kg/m^2^;
- Both sexes, aged 18 years or older;

The exclusion criteria for selecting subjects in the study were as follows:

- Previous diagnosis of type 1 or type 2 diabetes mellitus;
- Neurological, gastrointestinal, cardiac, renal, or pulmonary failure;
- History of cancer within the last 5 years;
- Acute illness.

**Fig. 1:**
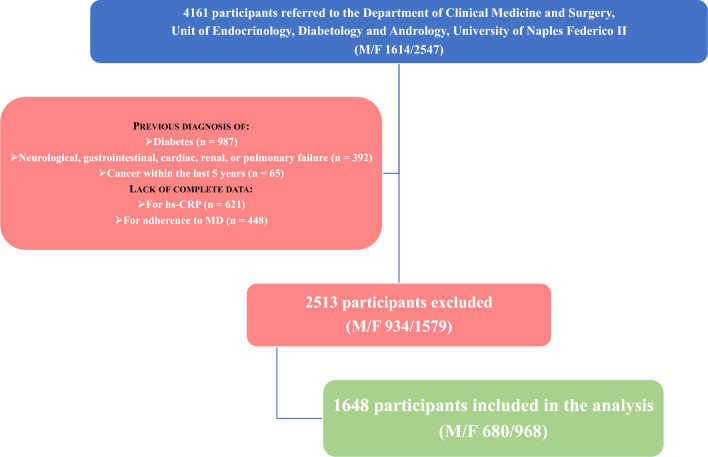
Flow chart of study participants

To be eligible for the study, all subjects had to undergo a medical examination, anthropometric evaluation, high-sensitivity C-reactive protein (hs-CRP) assessment, and evaluation of adherence to MD. Figure 1 shows the flow chart of study participants.

The study was conducted in accordance with the guidelines outlined in the Declaration of Helsinki, which provides ethical principles for medical research involving human subjects. Additionally, the Ethics Committee of the Federico II University of Naples reviewed the study procedures and granted a positive opinion on the study protocol (protocol no. 05/14).

### Anthropometric measurements

Anthropometric measurements were conducted between 8 and 10 a.m., following an overnight fasting period. A trained nutritionist performed these measurements using standardized techniques [15, 16]. During the assessment, participants were instructed to wear lightweight clothing and remove their shoes. To calculate the BMI of each subject, their height and weight were measured. Height (in meters) was determined using a wall-mounted stadiometer (Seca 711; Seca, Hamburg, Germany), with measurements recorded to the nearest 0.5 cm. Body weight (in kilograms) was measured using a calibrated balance beam scale (Seca 711; Seca, Hamburg, Germany), with measurements recorded to the nearest 0.1 kg. Furthermore, the waist circumference (WC) was measured to the nearest 0.1 cm using a non-stretchable measuring tape. The measurement was taken at the natural indentation of the waist or, if no natural indentation was visible, at a midpoint between the lower edge of the rib cage and the iliac crest. Abdominal obesity was defined as WC > 88 cm for women and > 102 cm for men.

### Physical activity and smoking habits

The methods employed in this study are consistent with those utilized in previous research. Physical activity levels were determined using a standardized questionnaire, which asked participants whether they engaged in at least 30 min of regular aerobic exercise *per* day (responding with either "yes" or "no"). Similarly, smoking habits were assessed using a standard questionnaire with "yes" or "no" responses. Individuals who had stopped smoking for a minimum of one year prior to the interview were categorized as "former smokers," while those who smoked at least one cigarette daily were classified as "current smokers." Participants who neither currently smoked nor had smoked within the past year were labeled as "non-current smokers." For the analysis, "former smokers" and "non-current smokers" were combined and referred to as "non-smokers."

### Adherence to the Mediterranean diet

Adherence to MD was assessed using the 14-item *Prevención con Dieta Mediterránea* (PREDIMED) questionnaire, which has been validated before [17]. As previously reported [18, 19], a trained nutritionist administered the questionnaire to all studied subjects during an in-person interview. Each item was assigned a score of 1 or 0, and the PREDIMED score was calculated based on the total scores [17]. Specifically, it was assigned a score of 1 for each of the following criteria met by the participants:

- Olive oil is the primary culinary fat and is consumed in a minimum quantity of 4 tablespoons *per* day.
- Consumption of at least two portions of vegetables *per* day.
- Consumption of at least 3 units of fruit or natural fruit juices *per* day.
- Restriction of daily consumption of red meat, butter, margarine and cream, to less than 1 unit *per* day.
- Consumption of at least 7 glasses of wine during meals within one week
- Consumption of at least 3 portions of legumes, fish, or shellfish in a week
- Limiting the consumption of commercial sweets or pastries to less than 3 times a week
- Restricting daily consumption of carbonated beverages to less than 1 unit *per* day
- Consumption of tree nuts at least once a week.
- Preference for chicken, turkey, or rabbit meat over red meat.
- Regular use of sofrito (a sauce made with tomato and onion, leek, or garlic, simmered with olive oil) at least twice a week.

A score of 0–5 indicated the lowest adherence to MD, a score of 6–9 indicated average adherence, and a score of ≥ 10 indicated the highest adherence to MD [17].

### High-sensitivity C-reactive protein (hs-CRP) assessment

The study assessed hs-CRP levels by collecting venous blood samples in the morning, between 8 and 10 a.m., after an overnight fast of at least 8 h. The hs-CRP levels were measured using a high-sensitivity nephelometric assay called the CardioPhase hs-CRP kit by Siemens Healthcare Diagnostics (Marburg, Germany). The assay had a lower detection limit of 0.01 mg/L and an intra- and interassay coefficient of variation (CV) of less than 7%. Based on guidelines from the Centers for Disease Control and Prevention and the American Heart Association, subjects were classified into three groups: low CVR (< 1.0 mg/L), intermediate CVR (1.0–3.0 mg/L), and high CVR (≥ 3.0 mg/L) [20].

### Statistical analysis

For continuous variables, the mean ± standard deviation (SD) was used to express the data, while categorical variables were presented as numbers (n) and percentages (%). The distribution of data was tested using the Kolmogorov–Smirnov test. Differences between female and male subjects in terms of sex, age, lifestyle habits, anthropometric measurements, inflammatory parameters, and nutritional parameters were assessed using the Student's independent *t*-test. The significance of differences in frequency distributions of categorical variables was determined using the chi-square (χ^2^) test. Proportional odds ratio (OR) models were employed to evaluate the association of female and male subjects with the dietary components of the PREDIMED questionnaire. Correlations between study variables were analyzed using Pearson's correlation coefficients for continuous variables. Receiver operating characteristic (ROC) curve analysis was conducted to determine the sensitivity, specificity, area under the curve (AUC), and cut-off values of the PREDIMED score in detecting the high CVR for both sexes. A *p*-value of less than 0.05 was considered statistically significant. The statistical analysis was performed using the Statistical Package for Social Sciences software version 26.0 (SPSS/PC; SPSS, Chicago, IL, USA), following standard methods.

## Results

Age, lifestyle habits, and anthropometric parameters of the study population of female and male study participants are displayed in Table 1. Females and males did not differ in age or BMI. Females were more physically active (p = 0.008) and less likely to smoke (p < 0.001) than males. The prevalence of overweight was significantly higher in males than females (p < 0.001); on the contrary, the prevalence of grade II obesity was significantly higher in females than males (p = 0.006). As expected, WC was significantly lower in females than males (p < 0.001).

**Table 1:** Age, lifestyle habits and anthropometric parameters of study population according to sex

Parameters	Females N = 968 (58.7%)	Males N = 680 (41.3%)	*p*-value
Age (Years)	33.81 ± 11.06	34.77 ± 11.31	0.088
Physical activity			χ^2^ = 7.15, ***p* = 0.008**
Yes	384 (39.7%)	225 (33.1%)	
No	584 (60.3%)	455 (66.9%)	
Smoking			χ^2^ = 67.82, ***p* < 0.001**
Yes	183 (18.9%)	253 (37.2%)	
No	785 (81.1%)	427 (62.8%)	
Anthropometric parameters			
BMI (kg/m^2^)	34.14 ± 7.43	33.77 ± 8.13	0.339
Normal weight (n, %)	154 (15.9%)	107 (15.7%)	χ^2^ = 0.01, *p* = 0.979
Overweight (n, %)	161 (16.6%)	161 (23.7%)	χ^2^ = 12.16, ***p* < 0.001**
Grade I obesity (n, %)	167 (17.3%)	132 (19.4%)	χ^2^ = 1.11, *p* = 0.291
Grade II obesity (n, %)	247 (25.5%)	133 (19.6%)	χ^2^ = 7.76, ***p* = 0.006**
Grade III obesity (n, %)	239 (24.7%)	147 (21.6%)	χ^2^ = 1.93, *p* = 0.164
WC (cm)	97.59 ± 22.26	111.13 ± 24.42	** < 0.001**
WC < cut off *	365 (37.7%)	281 (41.3%)	χ^2^ = 2.04, *p* = 0.153
WC > cut off *	603 (62.3%)	399 (58.7%)	

Data are expressed as number and percentage or mean ± SD. *BMI* Body Mass Index, *WC* Waist Circumference. A *p*-value in bold type denotes a significant difference (p < 0.05). * WC 88 cm and 102 cm for females and males, respectively

**Fig. 2:**
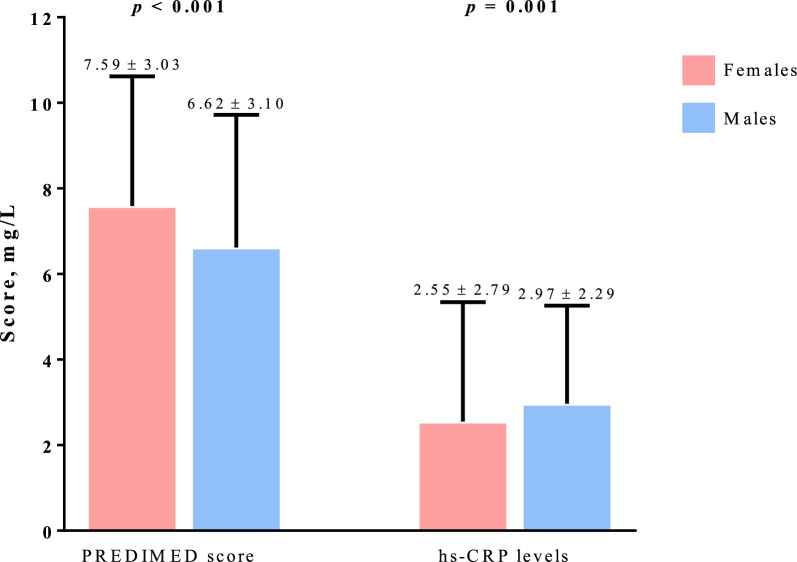
Sex differences in adherence to MD and hs-CRP levels. A *p-*value in bold type denotes a significant difference (*p* < 0.05). PREDIMED, PREvención con DIetaMEDiterránea; hs-CRP, High Sensitivity C Reactive Protein. Median values (minimum—maximum): PREDIMED score: 7 (1 – 14) *vs* 7 (1–14) females and males, respectively; hs-CRP levels: 1.80 (0.02–19.22 mg/L) *vs* 2.33 (0.90–17.70 mg/L) females and males, respectively

Figure 2 shows the sex differences in adherence to MD and hs-CRP levels. The PREDIMED score was significantly higher in females than in males (p < 0.001), while hs-CRP levels were significantly higher in males than in females (p = 0.001).

**Fig. 3:**
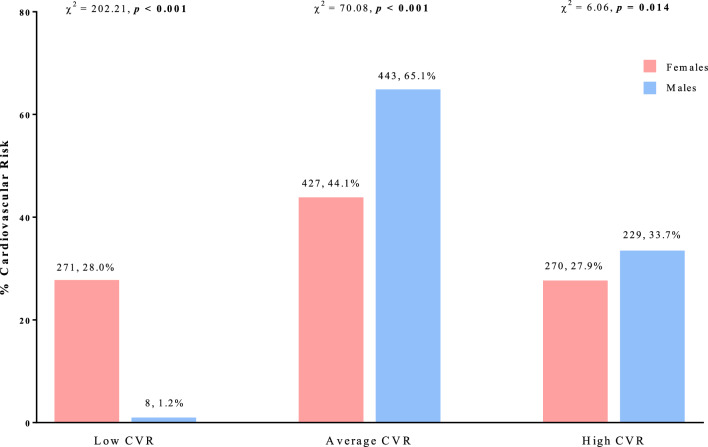
Cardiovascular risk categories assessed according to hs-CRP levels by sex. The % CVR was calculated based on sex. A *p-*value in bold type denotes a significant difference (*p* < 0.05). CVR, cardiovascular risk

Figure 3 shows the CVR categories assessed according to hs-CRP levels by sex. Females had a significantly lower CVR rate than males (p < 0.001). There were no significant differences in the intermediate risk rates between the two sexes.

Females consumed significantly more vegetables (p = 0.004), fruits (p = 0.001), legumes (p < 0.001), fish/seafood (p = 0.001), nuts (p < 0.001), and sofrito sauce (p < 0.001) and less quantity of EVOO (p = 0.025), butter, cream, margarine (p = 0.003), red/processed meats (p < 0.001), soda drinks (p = 0.001), red wine (p < 0.001) and commercial sweets and confectionery (p < 0.001) than males (Table 2).

**Table 2:** Response frequency of dietary components included in the PREDIMED questionnaire in study population according to sex

Questions of PREDIMED questionnaire	Females N = 968 (58.7%)		Males N = 680 (41.3%)		χ^2^	*p*-value
	n	%	n	%		
Use of EVOO as main culinary lipid	713	73.7	529	77.8	3.46	0.063
EVOO > 4 tablespoons	447	46.2	353	51.9	5.03	**0.025**
Vegetables ≥ 2 servings/day	530	54.8	323	47.5	8.13	**0.004**
Fruits ≥ 3 servings/day	607	62.7	371	54.6	10.66	**0.001**
Red/processed meats < 1/day	562	58.1	212	31.2	114.79	** < 0.001**
Butter, cream, margarine < 1/day	514	53.1	412	60.6	8.80	**0.003**
Soda drinks < 1/day	520	53.7	300	44.1	14.35	**0.001**
Wine glasses ≥ 7/week	324	33.5	421	61.9	129.28	** < 0.001**
Legumes ≥ 3/week	635	65.6	368	54.1	21.63	** < 0.001**
Fish/seafood ≥ 3/week	529	54.6	315	46.3	10.75	**0.001**
Commercial sweets and confectionery ≤ 2/week	541	55.9	229	33.7	78.28	** < 0.001**
Tree nuts ≥ 3/week	304	31.4	131	19.3	29.68	** < 0.001**
Poultry more than red meats	573	59.2	290	42.6	43.18	** < 0.001**
Use of sofrito sauce ≥ 2/week	550	56.8	246	36.23	67.33	** < 0.001**

Results are expressed as numbers and percentage. A *p*-value in bold type denotes a significant difference (*p* < 0.05). *PREDIMED* PREvención con DIetaMEDiterránea, *MD* Mediterranean Diet

**Fig. 4:**
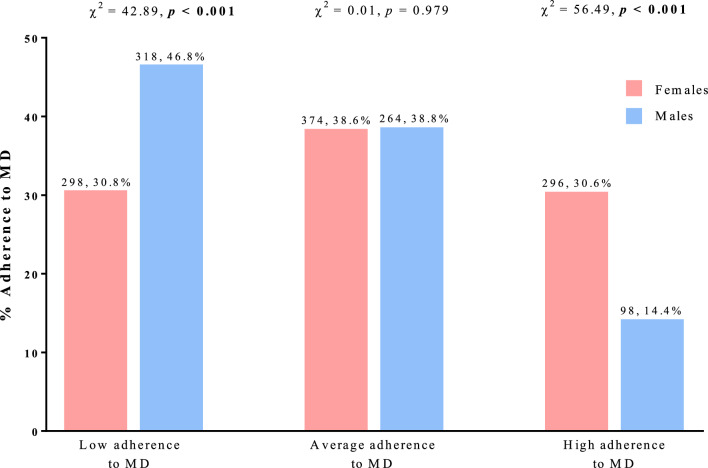
Adherence to MD according to sex. The % adherence to MD was calculated based on sex. A *p-*value in bold type denotes a significant difference (*p* < 0.05). *MD* Mediterranean Diet

Figure 4 shows adherence to MD according to sex. Females had significantly higher adherence to MD than males (p < 0.001). There were no significant differences in the intermediate adherence to MD between the two sexes.

In Table 3, the results of the bivariate proportional OR model performed to assess the association of female and male sex with dietary components of the PREDIMED questionnaire, PREDIMED score, and categories were summarized. Female sex was associated with the lowest consumption of EVOO > 4 tablespoons (p = 0.022), red/processed meats (p < 0.001), butter, cream, margarine (p = 0.003), soda drinks (p < 0.001), wine glasses (p < 0.001), commercial sweets and confectionery (p < 0.001) and use of sofrito sauce (p < 0.001) and the highest consumption of vegetables (p = 0.004), fruits (p = 0.001), legumes (p < 0.001), fish/seafood (p = 0.001), nuts (p < 0.001) and poultry (p < 0.001). Female sex was also associated with the highest score of adherence to MD (p < 0.001).

**Table 3:** Bivariate OR model to assess the association of gender with the dietary components included in PREDIMED questionnaire, PREDIMED score and categories

Questions of PREDIMED questionnaire	OR	*p*-value	95% IC	R^2^
Use of EVOO as main culinary lipid	0.80	0.055	0.634 – 1.010	0.002
EVOO > 4 tablespoons	0.79	**0.022**	0.653 – 0.967	0.003
Vegetables ≥ 2 servings/day	1.34	**0.004**	1.099 – 1.628	0.005
Fruits ≥ 3 servings/day	1.40	**0.001**	1.147 – 1.709	0.007
Red/processed meats < 1/day	3.06	** < 0.001**	2.486 – 3.757	0.069
Butter, cream, margarine < 1/day	0.74	**0.003**	0.604 – 0.899	0.006
Soda drinks < 1/day	1.47	** < 0.001**	1.207 – 1.791	0.009
Wine glasses ≥ 7/week	0.31	** < 0.001**	0.252 – 0.380	0.077
Legumes ≥ 3/week	1.62	** < 0.001**	1.323 – 1.976	0.013
Fish/seafood ≥ 3/week	1.39	**0.001**	1.147 – 1.700	0.007
Commercial sweets and confectionery ≤ 2/week	2.49	** < 0.001**	2.036 – 3.058	0.047
Tree nuts ≥ 3/week	1.92	** < 0.001**	1.518 – 2.424	0.019
Poultry more than red meats	1.95	** < 0.001**	1.599 – 2.380	0.026
Use of sofrito sauce ≥ 2/week	2.32	** < 0.001**	1.898 – 2.84	0.041
PREDIMED score	1.11	** < 0.001**	1.074 – 1.147	0.024
PREDIMED categories				
Low adherence to MD	0.51	** < 0.001**	0.413 – 0.620	0.026
Average adherence to MD	0.99	0.939	0.811 – 1.213	0.001
High adherence to MD	2.62	** < 0.001**	2.029 – 3.372	0.036

A *p-*value in bold type denotes a significant difference (*p* < 0.05). *PREDIMED* PREvención con DIetaMEDiterránea, *MD* Mediterranean Diet

Correlation analyses are reported in Table 4. In females, the PREDIMED score negatively correlated with age (p = 0.001), BMI (p < 0.001), WC (p < 0.001), and hs-CRP (p < 0.001). In males, the PREDIMED score positively correlated with age (p < 0.001) and negatively correlated with BMI (p < 0.001), WC (p < 0.001), and hs-CRP (p < 0.001). The correlation of the PREDIMED score with hs-CRP remained significant even after adjustment for age, BMI and WC, physical activity, and smoking in both sexes (both p < 0.001).

**Table 4:** Correlations of PREDIMED score with age, BMI, WC, and hs-CRP according to sex

Parameters	PREDIMED score							
	Females (n = 968)				Males (n = 680)			
	Simple		*Adjusted		Simple		*Adjusted	
	*r*	*p*-value	*r*	*p*-value	*r*	*p*-value	*r*	*p*-value
Age (years)	− 0.082	**0.011**	–	–	0.159	** < 0.001**	–	–
BMI (kg/m^2^)	− 0.744	** < 0.001**	–	–	− 0.583	** < 0.001**	–	–
WC (cm)	− 0.603	** < 0.001**	–	–	− 0.567	** < 0.001**	–	–
hs-CRP (mg/L)	− 0.508	** < 0.001**	− 0.257	** < 0.001**	− 0.457	** < 0.001**	− 0.270	** < 0.001**

A *p-*value in bold type denotes a significant difference (*p* < 0.05). *PREDIMED* PREvención con DIetaMEDiterránea, *BMI* Body Mass Index, *WC* Waist Circumference; *hs-CRP* High Sensitivity C Reactive Protein. *Adjusted for age, BMI and WC, physical activity, and smoking

Table 5 showed differences in age, BMI, WC, hs-CRP levels, and CVR between females and males, divided by adherence to MD. Females and males with low adherence to MD differed significantly in BMI (p < 0.001), WC (p < 0.001), hs-CRP levels (p = 0.026) and intermediate CVR (p = 0.002). Females and males with intermediate adherence to MD differed significantly in age (p = 0.012), WC (p < 0.001), hs-CRP levels (p < 0.001), low CVR (p < 0.001), and intermediate CVR (p < 0.001). Finally, females and males with high adherence to MD differed significantly in age (p < 0.001), WC (p < 0.001), hs-CRP levels (p < 0.001), low CVR (p < 0.001), and intermediate CVR (p < 0.001).

**Table 5:** Age, BMI, WC, and hs-CRP according to sex and adherence to MD

Parameters	Female subjects	Male subjects	*p*-value
**Low adherence to MD**	**N = 298 (48.4%)**	**N = 318 (51.6%)**	
Age (years)	34.98 ± 12.49	33.21 ± 12.69	0.082
BMI (kg/m^2^)	40.04 ± 5.25	37.79 ± 8.74	** < 0.001**
WC (cm)	112.15 ± 22.02	123.19 ± 26.58	** < 0.001**
hs-CRP (mg/L)	4.36 ± 3.44	3.79 ± 2.91	**0.026**
Low CVR (< 1.0 mg/L) (n,%)	5, 1.7%	3, 0.9%	χ^2^ = 0.07, p = 0.792
Intermediate CVR (1.0—3.0 mg/L) (n,%)	119, 39.9%	149, 46.9%	χ^2^ = 9.39, **p = 0.002**
High CVR (≥ 3.0 mg/L) (n,%)	174, 58.4%	166, 52.2%	χ^2^ = 0.03, p = 0.868
**Average adherence to MD**	**N = 374 (58.6%)**	**N = 264 (41.4%)**	
Age (years)	33.58 ± 10.90	35.72 ± 10.03	**0.012**
BMI (kg/m^2^)	35.09 ± 5.47	31.77 ± 5.31	** < 0.001**
WC (cm)	99.72 ± 17.66	104.81 ± 15.89	** < 0.001**
hs-CRP (mg/L)	2.31 ± 2.27	2.37 ± 1.27	0.701
Low CVR (< 1.0 mg/L) (n,%)	89, 23.8%	4, 1.5%	χ^2^ = 59.92, **p < 0.001**
Intermediate CVR (1.0—3.0 mg/L) (n,%)	198, 52.9%	199, 75.4%	χ^2^ = 32.20, **p < 0.001**
High CVR (≥ 3.0 mg/L) (n,%)	87, 23.3%	61, 23.1%	χ^2^ = 0.01, p = 0.961
**High adherence to MD**	**N = 296 (75.1%)**	**N = 98 (24.9%)**	
Age (years)	32.93 ± 9.57	37.27 ± 8.93	** < 0.001**
BMI (kg/m^2^)	26.98 ± 5.24	26.05 ± 3.57	0.103
WC (cm)	80.22 ± 14.79	89.04 ± 10.11	** < 0.001**
hs-CRP (mg/L)	1.03 ± 1.14	1.92 ± 0.60	** < 0.001**
Low CVR (< 1.0 mg/L) (n,%)	177, 59.8%	1, 1.0%	χ^2^ = 100.33, **p < 0.001**
Intermediate CVR (1.0—3.0 mg/L) (n,%)	110, 37.2%	95, 96.9%	χ^2^ = 103.02, **p < 0.001**
High CVR (≥ 3.0 mg/L) (n,%)	9, 3.0%	2, 2.1%	χ^2^ = 0.03, p = 0.867

A *p-*value in bold type denotes a significant difference (*p* < 0.05). MD, Mediterranean Diet; BMI, Body Mass Index; WC, Waist Circumference; hs-CRP, High Sensitivity C Reactive Protein; CVR, cardiovascular risk

**Fig. 5:**
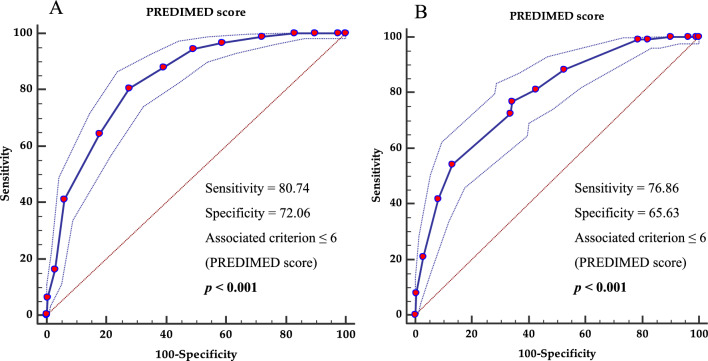
ROC for predictive values of PREDIMED score in detecting the high cardiovascular risk (hs-CRP ≥ 3.0 mg/L). A *p-*value in bold type denotes a significant difference (*p* < 0.05). *PREDIMED* PREvención con DIetaMEDiterránea

Two ROC analyses were performed to determine the cut-off values of adherence to MD predictive of high CVR (hs-CRP ≥ 3.0 mg/L) in female and male participants. A score of PREDIMED ≤ 6 could serve as a threshold for a significantly increased risk of high CVR both in females (p < 0.001, AUC 0.834, standard error 0.013, 95% CI 0.807 to 0.860, Fig. 5A) and males (p < 0.001, AUC 0.786, standard error 0.018, 95% CI 0.750 to 0.821, Fig. 5B).

## Discussion

The main finding of our study is that there was a sex-related adherence to MD. Of interest, females preferred the intake of vegetables, fruits, legumes, fish/seafood, nuts, and poultry more than red meats and sofrito sauce than males. Conversely, females had a lower tendency to consume large quantities of EVOO, red or processed meats, butter, cream and margarine, soda drinks, wine, and commercial sweets and confectionery than males. The different adherence to MD could also explain the different sex-related CVR represented by the hs-CRP levels in females compared to males. Indeed, the negative correlation between adherence to MD and hs-CRP levels remained significant even after adjustment for age, anthropometric measurements, physical activity, and smoking in both sexes.

Our findings agreed with previous studies reporting sex differences regarding dietary intake and eating behaviors [21]. Indeed, females are more likely to consume more fruit and vegetables, legumes, and whole foods and prefer more sweets and cakes. Males have a higher intake of fat- and protein-rich foods and drink more wine, beer, spirits, and sweet carbonated drinks, which makes them more predisposed to developing overweight and obesity. In a study carried out among college students in the US, sex-related differences in nutritional habits closely mirrored differences in weight status (with an increased prevalence of overweight and obesity in males) [22]. Data from an Italian study on adult subjects showed that females were more likely to follow a healthy lifestyle by consuming the recommended five portions of fruit and vegetables with a higher frequency daily than males [23]. This finding demonstrates that females are usually more motivated to follow a healthy eating pattern. The different sex-related nutritional habits could also be explained by the hormonal milieu. The estradiol/leptin ratio in the luteal phase has been reported to be positively associated with habitual sweets intake [24]. Females are also usually more worried about their body image, which could represent a deterrent to following an unhealthy lifestyle [25]. In this sense, a sex-related nutritional approach could be a tool used by females and males to reinforce their gender identity.

In addition, our study found that sex-related differences in adherence to MD could also partially explain the sex-related CVR. Indeed, it is well known that sex [26] and nutritional habits [27] could impact CVR. Recent epidemiological research has highlighted the effectiveness of dietary patterns, rather than individual nutrients, in studying eating habits and formulating therapeutic strategies for preventing and treating non-communicable diseases [28]. Adopting a healthy and personalized nutrition approach can significantly reduce CVR factors like obesity, diabetes, dyslipidemia, and high blood pressure, thereby playing a critical role in preventing the recurrence of chronic ischemic heart diseases [29]. Among dietary models, MD has been associated with a decreased risk of developing cardiovascular diseases, including coronary heart disease and myocardial infarction, as observed in the most recent epidemiological studies [30].

We found that in both sexes, adherence to MD was related to hs-CRP, a well-known marker of CVR. Although we identified the same cut-off values of PREDIMED score (≤ 6) to detect high CVR in both sexes, it is interesting to note that more than adherence to MD, food preference also plays a role in determining the sexes-related nutritional impact on CVR. We found that males were more likely to eat foods rich in saturated fats, which are well-known CVR factors. In fact, a prospective study carried out on 7038 participants at high CVR from the PREDIMED study from 2003 to 2010 documented 336 cardiovascular disease cases and 414 total deaths after 6 years of follow-up [31]. Interestingly, between extreme quintiles, higher saturated fatty acid and trans-fat intakes were associated with an 81% (HR 1.81; 95% CI 1.05 to 3.13) and 67% (HR 1.67; 95% CI 1.09 to 2.57) higher risk of cardiovascular disease [31]. In addition, we found that females were more likely to eat fruits and vegetables that are well known to have a protective effect against cardiovascular diseases. The Prospective Urban Rural Epidemiology (PURE), a prospective cohort study carried out in 135.335 individuals aged 35 to 70 years without cardiovascular disease from 613 communities in 18 low-income, middle-income, and high-income countries in seven geographical regions (North America and Europe, South America, Middle East, south Asia, China, southeast Asia, and Africa), highlights the role of fruit and vegetables in the context of cardiovascular diseases [32]. During a median of 7.4 years (5.5—9.3) of follow-up, 4784 major cardiovascular disease events, 1649 cardiovascular deaths, and 5796 total deaths have been recorded. Higher total fruit and vegetable intake were inversely associated with major cardiovascular disease, myocardial infarction, cardiovascular mortality, non-cardiovascular mortality, and total mortality in the models adjusted for age, sex, and center [32].

The clinical implications of our study are very significant and easy to use in an obesity outpatient clinic. Indeed, the knowledge of different sex-related preferences would allow the setting up of a tailored nutritional approach that, being more adherent to sex preferences, could also implement adherence.

Considering the observed differences between the sexes in adherence to MD, it is possible to customize MD to better suit the nutritional preferences and needs of each sex. For example, vegetable and fruit consumption can be adjusted, protein sources can be modified, fat sources can be adjusted, and preferences for drinks and sweets can be addressed to make MD “healthier” according to sex. It is also important to note that customization should be based on individual preferences, health conditions, and cultural factors, in addition to sex-related differences [33, 34]. Regarding alcohol consumption, MD suggests limited intake, primarily in the form of red wine, and associates it with cardiovascular health benefits [35, 36]. However, studies have raised concerns about the potential risks of alcohol consumption, even in moderate amounts [35]. Thus, it is crucial to consider individual needs, health conditions, and the potential risks associated with alcohol intake [37]. Individual factors, including sex, can influence alcohol consumption patterns, as observed in our study, where men consumed more alcohol than women, despite women showing higher adherence to MD. This highlights the complexity of dietary behaviors and the need for personalized considerations when evaluating alcohol intake. It is important to note that adherence to MD extends beyond the consumption of red wine for its potential antioxidant benefits [35]. The diet emphasizes a holistic approach that includes a wide variety of antioxidant-rich foods, such as fruits, vegetables, whole grains, and healthy fats. These components contribute significantly to the overall health benefits associated with MD.

In addition, we have identified a cut-off of the PREDIMED score that could be used as a tool to screen subjects at high CVR in both sexes that may need a more intensive therapeutic strategy and follow-up. Consulting with nutritionists or healthcare professionals can provide personalized guidance and support in implementing a customized MD approach.

The limit of the study is mainly related to the cross-sectional design, which does not allow any conclusion on causality. The use of a questionnaire (PREDIMED) to investigate adherence to MD was also another limit. Further scientific investigation is warranted to ascertain whether there would be any discrepancy in administering the questionnaire by trained nutritionists or by the subjects themselves. However, in order to reduce any bias, the questionnaire was not self-reported, and it was administered by the same nutritionist. In addition, despite the evidence of sex differences in body composition and total calorie intake, our study did not include such data, which are known to be CVR factors [38, 39]. This was done deliberately to focus on adherence to the MD and sex differences in order to delve deeper into personalized dietary interventions. However, for a more comprehensive understanding of the interaction between dietary patterns, total calorie intake, body composition, and cardiovascular health based on sex, future research should consider these variables. Finally, while our study focused on the non-modifiable factor of sex, we acknowledge that educational and social variables were not considered but can significantly influence eating behavior [33]. By taking into account the impact of cultural traditions and societal pressures and providing information on the ethical aspects of food choices, it is possible to foster a shift towards more sustainable and health-conscious eating patterns. For example, encouraging men to explore alternative protein sources, such as plant-based proteins or leaner animal options, can contribute to improved health outcomes and reduced environmental impact. However, it is important to recognize that changing dietary behaviors is a complex process influenced by various factors. Therefore, a multifaceted approach involving education, policy changes, and supportive environments is needed to create an environment that promotes healthier and more sustainable food choices for both females and males.

This study also has some strengths. The single-center study design of this research, although it could represent a selection bias due to the limits on the generalizability of our findings, allowed us to increase the homogeneity of the sample. In particular, all participants included came from the same geographical area, thus possibly sharing an overall similar food availability. Moreover, we adjusted our data for different confounding factors that might have an influence on sex differences in adherence to MD, including age, BMI, WC, physical activity, and smoking.

This novel association might uncover a further potential clinical application for MD based on the evaluation of sex differences relating this healthy pattern of nutrient intake to health outcomes on CVR sex-specific.

## Conclusions

In conclusion, we demonstrated that females have a higher adherence to MD than males, and this difference could contribute to explaining the different sex-related CVR; graphical abstract. Females prefer the intake of vegetables, fruits, legumes, fish/seafood, nuts, poultry more than red meats, and sofrito sauce than males. Conversely, females have a lower tendency to consume large quantities of EVOO, red/processed meats, butter, cream and margarine, soda drinks, wine, and commercial sweets and confectionery than males. The different adherence to MD could also explain the different sex-related CVR represented by the lower hs-CRP levels in females compared to males. A cut-off of PREDIMED score < 6 was associated with an increased risk of having cardiovascular diseases in both sexes. Thus, the PREDIMED score alone is not sufficient to accurately stratify the CVR of individuals of both sexes as it lacks sex specificity. Therefore, a detailed assessment of individual foods within the MD is necessary for clinical practice to assess the sexes-related nutritional impact on cardiovascular disease. Thus, it is crucial to have detailed nutritional information and the expertise of a nutritionist to determine which foods should be increased or reduced in a sex-specific manner to reduce CVR. Although these results are encouraging, randomized clinical trials are needed to investigate if a sex-related nutritional approach could implement lifestyle interventions in managing obesity and obesity-related CVR effectively.

## Data Availability

The datasets used and/or analyzed during the current study are available from the corresponding author on reasonable request.
